# Colorimetric Grading Scale Can Promote the Standardization of Experiential and Sensory Evaluation in Quality Control of Traditional Chinese Medicines

**DOI:** 10.1371/journal.pone.0048887

**Published:** 2012-11-07

**Authors:** Jia-bo Wang, Ling-na Zeng, Qing-ce Zang, Qian-feng Gong, Bao-cai Li, Xue-ru Zhang, Xiao-hui Chu, Ping Zhang, Yan-ling Zhao, Xiao-he Xiao

**Affiliations:** 1 China Military Institute of Chinese Medicine, 302 Military Hospital, Beijing, China; 2 Faculty of Life Science and Technology, Kunming University of Science and Technology, Kunming, China; 3 Pharmacy College, Jiangxi University of Traditional Chinese Medicine, Nanchang, China; 4 Integrative Medicine Center, 302 Military Hospital, Beijing, China; College of Tropical Agriculture and Human Resources, University of Hawaii, United States of America

## Abstract

Experiential and sensory evaluation is an ancient method that remains important in the current quality control system of Traditional Chinese Medicines (TCMs). The process is rapid and convenient when evaluating the quality of crude materials in TCM markets. However, sensory evaluation has been met with skepticism because it is mainly based on experience and lacks a scientific basis. In this study, rhubarb was selected to demonstrate how color-based sensory evaluation could differentiate the quality of herbal medicines objectively. The colors of the rhubarb samples, expressed as RGB values, were obtained from different parts and forms of the plant, including the plant’s surface, fracture surface color, and a powdered form with or without treatment with a color-developing reagent. We first divided the rhubarb samples into three grades based on the total content of five hydroxyanthraquinone derivatives, the major pharmacological components in rhubarb. Then, a three-layer back-propagation artificial neural network (BP-ANN), calibrated with selected training samples, was used to correlate the quality of the rhubarb with its color. The color of the rhubarb powder after coloration attained the highest accuracy (92.3%) in predicting the quality grade of the test samples with the established artificial neural networks. Finally, a standardized colorimetric grading scale was created based on the spatial distribution of the rhubarb samples in a two-dimensional chromaticity diagram according to the colors of the powdered rhubarb after color enhancement. By comparing the color between the scale and the tested samples, similar to performing a pH test with indicator paper, subjects without sensory evaluation experience could quickly determine the quality grade of rhubarb. This work illustrates the technical feasibility of the color-based grading of rhubarb quality and offers references for quantifying and standardizing the sensory evaluation of TCMs, foods and other products.

## Introduction

Experiential and sensory evaluation is a rapid and effective evaluation method that has been used widely in various fields [1]–[3]. The technique has been used for more than 30 years in the food industry as a quality control test, characterizing the taste and colors of foods, and is becoming increasingly important in food markets [4]–[6]. A set of unique methods of sensory evaluation have been developed as Traditional Chinese Medicines (TCMs) evolves. Traditional sensory evaluation is based primarily on the human senses to assess shape, size, texture, color, odor and taste. Sensory evaluation remains one of the most effective methods to assess the raw materials used in TCM. Compared with modern quality control methods that involve spectroscopy, chromatography, mass spectrometry and cutting-edge biotechnology, sensory evaluation is convenient, fast and effective [7]. Nevertheless, these high-tech modern methods have been greatly researched, whereas the conventional sensory evaluation methods interest few modern researchers. In China, quality evaluation based on sensory characteristics is widely used in the TCM markets. There are some commercial specifications of TCMs conventionally accepted by the growers, medicinal vendors, TCM physicians and consumers. Also there are *Dao-di* herbs (good quality herbs produced in the native areas) conventionally accepted by practitioners to differentiate the quality of TCMs. Those commercial specifications and the concept of *Dao-di* herbs are generally defined by a set of specific morphological features. Growers of medicinal herbs typically select a superior species or variety to cultivate based on those specific morphological features. Similarly, medicinal vendors classify and price TCMs based on different specifications, and consumers primarily evaluate the quality of a TCM on the basis of sensory characteristics [8]. The repeatabilities of the experiential and sensory assessment by practitioners for the TCMs, like rhubarb and *Coptis chinensis*, etc., have been demonstrated in our previous studies [9], [10]. Although traditional sensory evaluation has been regularly applied in China, there are some disadvantages. One concern is whether the sensory evaluation has a scientific foundation and chemical basis. A second issue is how the practice can be applied by people with little sensory evaluation experience, specifically users in the Western world.

**Figure 1:**
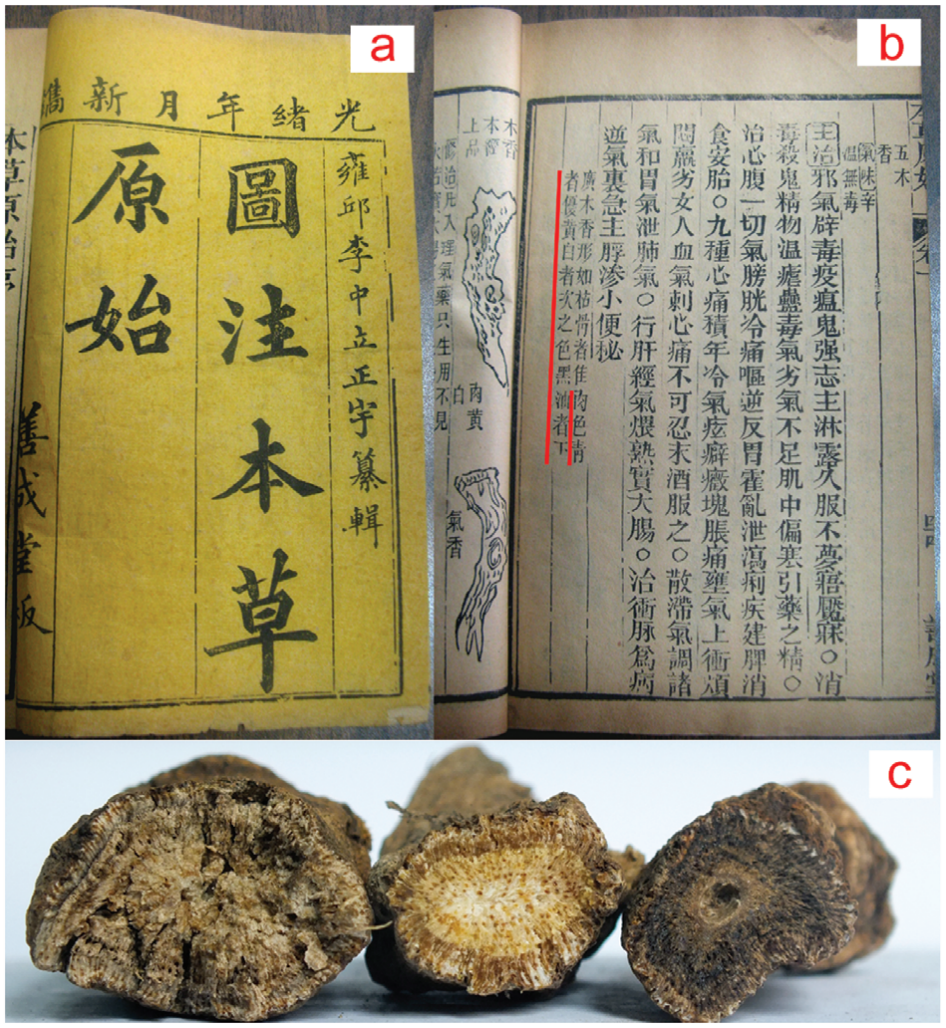
The herbal classic *Origin of the Materia Medica* (*Ben Cao Yuan Shi*) describes the color-based quality evaluation of Chinese herbal materials. a) *Origin of the Materia Medica*, written by Zhong-li Li in 1612 during the Ming Dynasty, has been considered the first of the herbal classics in China, which described the sensory characteristics for identification of commercial herbal materials (the medicinal parts of the plants) in detail rather than the plant morphology. b) The original description in the book for the color-based quality evaluation of *Aucklandia* root (*Aucklandia lappa* Decne, *muxiang* in Chinese). *Aucklandia* root is an herb that has been commonly used to treat gastroenterological diseases in China for over two thousand years. c) Photographs of the *Aucklandia* root samples in different color. The quality of *Aucklandia* root can be placed into three grades based on the color of its fracture surfaces: cadet blue denotes superior quality (left); yellowish-white indicates ordinary quality (middle); and an oleaginous black color suggests inferior quality (right).

Color is one of the most important characteristics in sensory evaluation for TCMs. Some ancient Chinese herbal medicine literature, such as the *Origins of the Materia Medica* (*Ben Cao Yuan Shi*), emphasize the importance of color as an identifier of high quality herbal materials (Figure 1). For example, an *Aucklandia* root (*muxiang* in Chinese) that is cadet blue in color is considered to be superior, mid-grade if yellowish-white, and inferior in quality when characterized by an oleaginous black color [11]. The correlation between color and chemical composition, however, is not well studied. Furthermore, sensory evaluation relies greatly on personal experiences, which are difficult to quantify and standardize. Thus, its application has been restricted. To our knowledge, no previous quantification and standardization of color evaluation for Chinese herbal medicine has been reported.

Rhubarb, an ancient and one of the best known Chinese herbal medicines, has been recognized for centuries in traditional medicine for its pharmacological properties, including its purgative [12], nephric protecting [13], [14], liver protecting [15], antimicrobial and hemostatic properties [16]. The major pharmacological components in rhubarb are five hydroxyanthraquinones (HAQs) – aloe-emodin, rhein, emodin, chrysophanol and physcion – which are also quality control markers in the Chinese pharmacopoeia [17]. The quality of rhubarb correlates directly with the content of the five HAQs. Rhubarbs of different grades usually exhibit different colors, such as dark red, brown yellow or yellow. According to traditional sensory evaluation, rhubarbs of good quality are often bright yellow and those of poor quality were often dark yellow or brown. The objectivity and reproducibility of sensory evaluation for rhubarb have been demonstrated using the Delphi method [9]. In this study, rhubarb has been selected as a model drug for its representative color in sensory evaluation.

**Figure 2:**
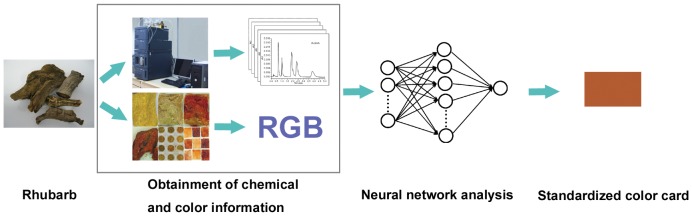
The flowchart for this study.

The overall flowchart of this study is shown in Figure 2. First, we divided the rhubarb samples into three grades based on the total content of the five HAQs. Second, we used the three-layer back-propagation artificial neural network (BP-ANN) to find relationships between the quality and the color of the samples. Finally, we defined a colorimetric grading scale for the quality of rhubarb. By comparing the color of a sample with this scale, similar to matching the color of litmus paper with a color scale to ascertain pH, customers can quickly determine rhubarb quality. To investigate which kind of sample produced the best colors to use for grading rhubarb, colors from the plant surface, fracture surface color and powder, with or without coloration by a color-developing reagent, were analyzed. Through this work, we illustrated the feasibility of color-based grading for rhubarb and developed a practical tool, a standardized colorimetric grading scale, to classify the rhubarb. Furthermore, we provided some references for the quantification and standardization of sensory evaluation of TCMs.

## Materials and Methods

### Materials

Thirty-four batches of rhubarb were collected from various sources in China (Table 1). All of the voucher specimens were deposited at the China Military Institute of Chinese Materia Medica. The samples were identified by Professor Xiao-He Xiao as the dried roots and rhizomes of *Rheum palmatum* L., *Rheum tanguticum* Maxim. ex Balf. and *Rheum offcinale* Baill. The chemical reagents were analytical grade and obtained from the Beijing Chemical Factory (Beijing, China).

**Table 1:** RGB values of the color of the surface, the fracture surface and powder with or without further color development.

No.	Collection place	Grade	Surface color				Fractured surface color							Powder color						
			Without coloration				Without coloration			With coloration				Without coloration				With coloration		
			R	G	B	R		G	B	R	G	B	R		G	B	R		G	B
1	Li, Gansu	2nd	176	141	42	162		142	78	125	38	34	195		148	38	203		85	26
2	Li, Gansu	2nd	159	132	37	152		151	116	127	55	37	154		123	33	167		74	46
3	Li, Gansu	2nd	181	150	47	166		151	100	147	52	31	175		135	27	196		83	35
4	Li, Gansu	2nd	110	82	32	153		154	101	136	54	31	123		88	33	181		79	43
5	Li, Gansu	3rd	90	72	37	202		181	112	198	79	34	144		113	43	216		120	44
6	Li, Gansu	1st	149	125	33	165		150	85	144	80	31	171		131	34	240		163	69
7	Li, Gansu	1st	102	86	47	105		91	42	44	42	44	118		90	31	173		85	46
8	Dali, Yunnan	2nd	146	115	36	155		154	103	119	57	39	166		134	27	233		159	63
9	Dali, Yunnan	3rd	84	70	37	115		107	70	80	42	43	116		87	29	233		172	92
10	Dali, Yunnan	3rd	127	97	34	162		170	130	146	74	28	150		112	12	231		144	46
11	Gansu	2nd	103	86	44	117		107	55	92	47	39	119		92	31	197		114	61
12	Qinghai	1st	128	95	28	126		93	31	94	47	33	132		101	21	143		64	45
13	Xining, Qinghai	2nd	143	112	37	145		142	110	126	57	35	174		125	25	182		95	41
14	Fengjie, Chongqing	3rd	104	84	33	112		109	47	57	53	39	103		81	36	180		101	49
15	Xining,Qinghai	1st	128	97	33	141		140	119	103	53	45	148		101	32	128		58	51
16	Gansu	3rd	129	95	30	158		156	86	115	64	31	145		105	17	188		85	41
17	Shanxi	2nd	142	114	42	124		111	77	132	50	44	170		125	41	181		89	43
18	Gansu	3rd	108	92	53	114		110	42	88	56	36	108		79	27	166		83	46
19	Tibet	1st	106	83	44	127		120	61	101	54	47	126		86	18	156		58	42
20	Shiqu, Sichuan	1st	104	88	47	151		140	92	165	77	26	133		94	19	192		80	32
21	Saichuan, Sichuan	1st	113	95	49	156		115	33	112	44	46	158		112	21	200		91	33
22	Changdu, Tibet	2nd	110	84	33	104		78	50	65	34	38	110		79	24	124		60	55
23	Gansu	2nd	128	109	45	153		151	112	134	67	37	145		95	24	196		99	43
24	Tibet	2nd	111	93	56	146		138	73	115	58	41	133		103	27	198		84	40
25	Beichuan, Sichuan	2nd	82	65	34	139		138	86	105	59	42	111		81	25	166		65	44
26	Liangshan, Sichuan	2nd	99	76	36	164		141	85	157	50	31	143		98	23	202		84	31
27	Guizhou	3rd	109	85	31	117		100	35	67	47	43	109		76	25	185		89	44
28	Yaan, Sichuan	2nd	123	91	26	166		164	135	154	62	33	151		110	22	198		74	36
29	Changdu, Tibet	2nd	93	72	35	140		110	46	107	49	38	125		88	19	182		79	48
30	Gansu	3rd	123	93	28	137		125	49	107	72	38	115		78	19	177		95	82
31	Qinghai	1st	121	82	22	128		111	39	96	53	42	120		87	24	158		70	51
32	Sichuan	2nd	141	102	25	146		122	48	115	45	36	141		100	20	192		81	40
33	Gansu	3rd	115	91	29	129		111	49	100	76	40	125		96	27	188		107	63
34	Qinghai	3rd	102	87	38	108		105	56	64	56	37	123		102	25	213		143	87
	R*		0.192	0.128	−0.020	0.014		−0.101	−0.004	0.056	−0.265	0.131	0.199		0.092	−0.181	−0.388		−0.460	−0.341

* The pearson’s correlation coefficient between the color data (R, G or B values) and the total content of HAQs in the samples.

### Ethics

No specific permits were required for the described field studies. The aforementioned locations are neither privately-owned nor protected by the Chinese government. And there is no endangered or protected species involved in the specific locations where we collected the rhubarb samples.

### Camera System

Images were acquired using a camera system equipped with a standard lightbox, camera, computer, ZoomBrowser EX software, data lines and digital camera (PowerShot G5 Pro 5.0 Megapixel, Canon Inc., Nagasaki, Japan). The standard lightbox consists of a steel framework, fluorescent lamp (LUMILUX, FH14W/865HE, OSRAM Company, München, Germany), ballast and shading-cloth.

### Optimization of Shooting Conditions

Exposure time, aperture size, and light sensitivity were optimized to obtain clear images. The optimal camera performance parameters were determined by varying exposure time (1/4, 1/6, 1/8, 1/10, 1/13, 1/15, 1/20, 1/30, 1/40, 1/60, 1/80, 1/100), aperture size (8.0, 7.1, 6.3, 5.6) and ISO light sensitivity value (50 and 100). The results showed that the clearest image with well-balanced brightness and contrast was acquired using the following conditions: an exposure time of 1/10 second, an aperture size of 8.0 and an ISO light sensitivity value of 50.

### Sample Coloration and Obtainment of RGB Values

Powder coloration: The powdered rhubarb samples of 0.3 g were weighed respectively and then dispersed into a 96-well plate which is generally used in cell culture and molecular biology experiments. Then an aqueous solution of 0.5% NaOH (0.6 ml) was added to each well to develop the color of the sample. After coloration, pieces of square filter paper (1.5×1.5 cm) were then inserted into the colored powder to absorb the rhubarb solution in the wells, and the filter papers were transferred to the standard lightbox for immediate imaging.

Fractured surface coloration: NaOH solution (0.5%, 0.2 ml) was sprayed onto the fractured surface of each sample of rhubarb. These samples were photographed immediately in the lightbox.

**Figure 3:**
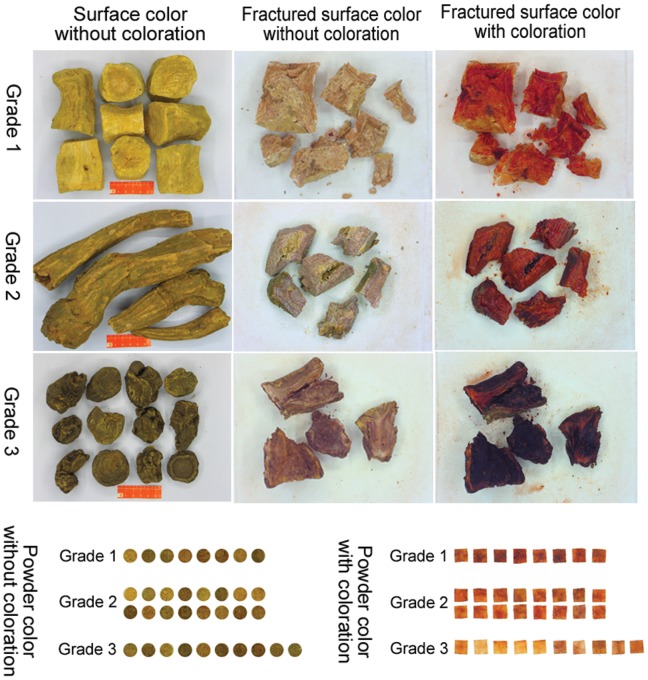
Representative images of rhubarb samples of different grades.

All samples, with or without coloration, were placed in the standard lightbox, and photographs were taken using the optimized shooting conditions described above. Some representative images are shown in Figure 3. All images were saved in the.jpg format. The RGB values were extracted from the images using Photoshop CS4 (Adobe Systems Inc., USA) image processing software.

### BP Neural Network Analysis

**Figure 4:**
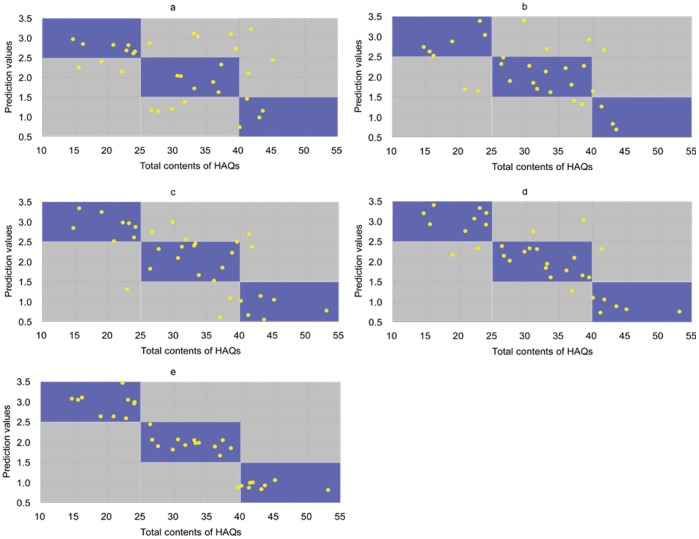
Color-based predictions of the grades of rhubarb samples from the BP network. All the thirty-four samples were counted here, including the training set and the test set. The blue areas indicate correct predictions, and the gray areas indicate incorrect predictions. a, Using the color from the surface of rhubarb without additional coloration as the input parameter, the percentage of correct predictions was 50.0% (17 correct predictions in 34 samples). b, When the colors of the fracture surfaces without development were input, the percent of correct predictions was 64.7% (22 correct predictions in 34 samples). c, When the color-enhanced fracture surfaces were analyzed, the percent of correct predictions was 73.5% (25 correct predictions in 34 samples). d, Using the powder without coloration as the input, the percent of correct predictions was 79.4% (27 correct predictions in 34 samples). e, Analysis of the powder after color development yielded predictions that were 91.2% accurate (31 correct predictions in 34 samples).

A BP neural network with one input layer, one hidden layer, and one output layer was established in this study. According to the experimental results, the RGB values were set as three of the evaluation parameters. In this experiment, there were seven initial nodes in the hidden layer, and the number of nodes was defined to be 2N+1, where N is the number of input layer nodes. In the output layer, three nodes were designated to represent the three grades of rhubarb. The hidden layer and input vectors were connected, and the output layer and input vectors were not. In addition, the training sample set and the desired values were normalized with *Premnmx* from the Matlab toolbox. Finally, the BP network was established using the *Newcf* function and the transfer function of neurons in each layer was *tansig* and *purelin*. The network training function was *trainParam*. After training, the grading standards were saved in the network, and the BP neural network was able to produce a prediction. Although the determined sample grades are discrete numbers (1, 2, or 3) in the training process, the predicted values are continuous real numbers. The grade of an inputted sample could be designated based on the predicted value. For example, if the output value is in the range of (0.51–1.50], the sample should be designated as the grade 1 (Figure 4). Additionally, for the grade 2 and grade 3, the ranges are designated as (1.51–2.50] and (2.51–3.50], respectively. In the results predicted by the BP network, some output values were not in the range of (0.51–3.50]. These numbers were considered incorrect predictions.

The process can be summarized as follows:

i. Input the quality evaluation parameters of the training samples into the network input nodes.
ii. Create the BP neural network.
iii. Select proper training functions and parameters.
iv. Output the results from the prediction to the output nodes of the network.
v. Input the three evaluation parameters for the samples.
vi. Proceed through the Matlab simulation.
[pn,meanp,stdp,tn,meant,stdt] = prestd(p,t); % normalizing
% establish neural network
net  =  newcf (minmax(ptr), [21 1], {‘tansig’ ‘purelin’}, ‘traingdx’, ‘learnsom’);
% train the neural network
net.trainParam.epochs = 4500;
net.trainParam.goal = 0.000001;
net.trainParam.show = 40;
net.trainParam.lr = 0.05;
[net,tr] = train (net,ptr,ttr,[],[],val,test);
a  =  poststd(an,meant,stdt); % reverse normalization
% end

### Color Conversion for Rhubarb Samples

Because the RGB color space consists of three dimensions, it is difficult to use RGB information to summarize the distribution of the rhubarb samples in each quality grade. To distinguish the lower and upper thresholds for grading different rhubarb samples, the RGB values were transformed into the CIE1931 XYZ color space, where the values can be normalized and plotted in a two-dimensional CIE1931xy chromaticity diagram. The formulas used to convert between the CIE XYZ and RGB color spaces are well documented [18], [19]. The CIE XYZ color space is deliberately designed so that the Y parameter is a measure of the brightness or luminance of a color. The chromaticity of a color is then specified by the derived parameters x and y, two of the three normalized values which are functions of the three tristimulus values X, Y, and Z.
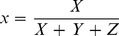
(1)

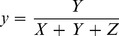
(2)

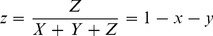
(3)


The derived color space specified by x and y can represent all of the chromaticities visible to the average person within a two-dimensional chromaticity diagram, which is a widely used tool to specify colors [18].

### Design of the Colorimetric Grading Scale

**Figure 5:**
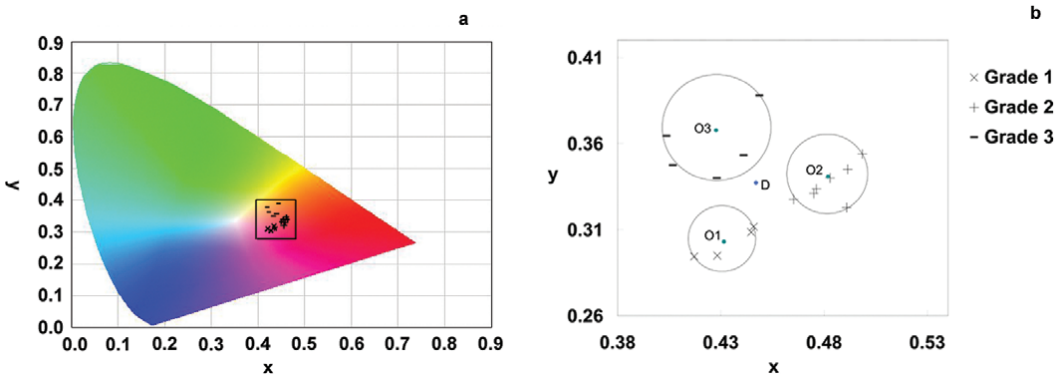
The distribution of the rhubarb samples in each grade in the CIE1931xy chromaticity diagram. a, The overall distribution of the samples in the two-dimensional coordinates overlaying on the CIE1931xy chromaticity diagram. The samples in the grade 1 mainly distribute in the lower left region favoring red-purple; the samples in the grade 2 distribute in the right region toward orange; and the samples in the grade 3 distribute in the upper left region tending toward yellow. b, Schematic diagram describing the geometric calculations for the segmentation points. The diagram represented the magnified region in the panel a.

To establish the color scale for colorimetric grading, we assumed that rhubarb samples of different quality would distribute randomly in a circle centered at the expected color values (points *O_1_*, *O_2_* and *O_3_*, shown in Figure 5a) for the samples. These values are typically within the radius of the maximum distance from the center in the chromaticity diagram. The centroid (point *D*) of the triangle that consists of the points *O_1_*, *O_2_* and *O_3_* can be assigned as the division point that divides the samples into the different quality grades.

## Results

### Quality Grade Assignment for the Rhubarb Samples

Before the BP-ANN analysis, the rhubarb samples were divided into three grades according to the total content of the five HAQs (*T_HAQs_*) based on the following definitions: grade 1, *T_HAQs_*≥40.00 mg g^−1^, grade 2, 25.00<*T_HAQs_*<40.00 mg g^−1^, and grade 3, *T_HAQs_* ≤25.00 mg·g^−1^ (Table S1).

### Color Quantization into RGB Values for the Rhubarb Samples

Images of a sample were captured in the standard lightbox and under natural light at different times (morning, noon, afternoon, evening) during the day using the optimized shooting conditions described above. Table 2 shows the relative standard deviations (RSD) of the R, G and B channels, which were 10.24%, 10.22% and 11.75%, respectively, when shooting under natural light at different time points. In contrast, the RSD for the R, G and B values were 0.94%, 1.13% and 1.81%, respectively, when shooting in the lightbox. The lighting conditions provided by the lightbox enable good reproducibility when acquiring images for color information. In this study, we acquired different colors of the herb, including the outside surface, a fracture surface, and a powder, with or without coloration by a developing reagent (Figure 3). The RGB values from the 34 rhubarb samples are shown in Table 1. The data of surface color with coloration were not listed and excluded from the research because the data varied greatly in different zones of the herb surface.

**Table 2:** The reproducibility of the RGB measurements.

Experiment	Under natural light			In standard lightbox		
	R	G	B	R	G	B
1	116	102	57	114	103	56
2	117	103	57	114	103	56
3	117	103	57	114	102	56
4	121	106	51	112	100	54
5	122	106	57	113	102	54
6	120	106	54	111	101	55
7	119	105	54	112	101	53
8	115	98	51	112	100	54
9	123	100	49	112	100	55
10	110	96	49	112	101	55
11	87	77	42	112	101	55
12	105	93	51	114	102	55
13	110	98	53	113	102	54
14	92	89	39	112	101	54
15	96	75	41	111	99	53
RSD (%)	10.24	10.22	11.75	0.94	1.13	1.81

### Neural Network Establishment and Prediction

The neural network was established firstly by processing the training set, and then the other samples were taken as the test set to assess the validity of the predictions and results from the network. We assumed the samples containing intermediate amount of HAQs in a certain quality grade are representative for the whole of the grade. So those samples of *T_HAQS_* in the intermediate range (20% less than or greater than the upper or lower limit of each quality grade) were chosen as the training samples: The grade 1 consisted of samples 6, 7, 12, 15, 19, 20 and 21; the grade 2 contained samples 1, 2, 3, 4, 8, 11 and 17; and the grade 3 were samples 5, 9, 10, 14, 16, 27 and 30. After establishment of the BP network, the other thirteen samples out of the training set were input as test samples and predicted. The prediction accuracy by the BP network diversified depending on which form of color of the herb was examined. The prediction accuracy values of each kind of color were: 38.5% (5 correct predictions in 13 test samples) for the surface color, 92.3% (12 correct predictions in 13 test samples) for the powder color after coloration and 69.2% (9 correct predictions in 13 test samples) for the other three kinds of color. In addition, the predictions for the former training samples were also counted and combined with the results of the test samples. The prediction values were plotted with the contents of the five HAQs in Figure 4. It could be found that the color of the rhubarb powder after coloration attained the lowest wrong prediction of the quality grade for all the samples.

### Calculation of the Boundaries that Differentiate Grades of Rhubarb

**Figure 6:**
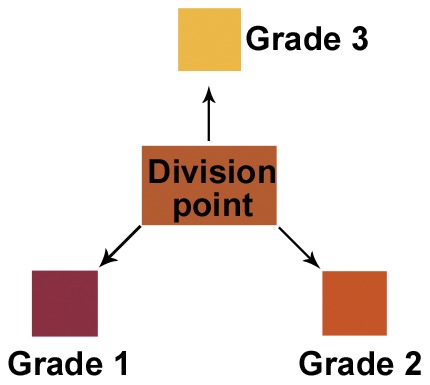
Differences between the color at the division point and the colors of the mathematical expectation points for each grade.

The sixteen representative samples (sample 12, 15, 19 and 31 in the grade 1; sample 1, 3, 4, 24, 26, 28 and 32 in the grade 2; and sample 9, 10, 14, 33 and 34 in the grade 3) showing the least overlap in their distribution in the chromaticity diagram (i.e., distributed approximately 80% of the possible maximum distance from the center of each quality grade) were selected to determine the boundaries for differentiating the rhubarb grades. The acquired RGB values of the representative samples were converted to values within the CIE 1931xy color space and then plotted in the chromaticity diagram (Figure 5a). The distribution of the three grades of the rhubarb samples separated into distinct regions. According to the calculations described above, the mathematical expectation points (*x*, *y*) of each grade are *O_1_* (0.4315, 0.3031), *O_2_* (0.4819, 0.3408) and *O_3_* (0.4277, 0.3677). The division point *D* (*x*, *y*) is defined as the geometric center of the three mathematical expectation points calculated for the three grades and occurs at (0.4470, 0.3372). Figure 5b shows that the rhubarb samples in each grade distribute away from the division point (*D*) in three directions toward a different color category. The grade 1 tends toward the red-purple region; the grade 2 to the orange region; and the grade 3 to the yellow region. Thus, how the samples distribute can be used to determine the grade of an unknown rhubarb sample, specifically by comparing the color of the unknown with a colorimetric grading scale created based on the color of the division point (*D*). Corresponding ribbons was drawn using the software from the RGB values of the division point (*D*) and the mathematical expectation points (*O_1_, O_2_, O_3_*) for each grade (Figure 6).

## Discussion

Because of the convenience and speed of sensory evaluation compared with the laboratory analyses of chemical content, the desired color-based grading method would help in assessing the quality of rhubarb in the TCM marketplace. The approach for obtaining the color information is important for sensory evaluation. A colorimeter is a device that is commonly used to measure the color components of solid samples. In our preliminary experiment, we found that measuring the color of rhubarb by colorimeter was difficult because the plant’s surface or fracture surface was not smooth and uniformly colored, which decreased the repeatability of the results. Unfortunately, most herbal medicines have various shapes that affect reproducibility. In this study, we used a digital camera to capture images of samples and extracted the color data from these images using Photoshop (Adobe Corporation, USA). Through this approach, the color data of the herbs are consistent with how colors are perceived by human vision. The RGB (red, green, blue) color model is one of the most widely used of the color systems. An additive model in which red, green, and blue light are summed to produce a broad array of colors, the model includes almost all of the colors that can be perceived by the human eye. In this study, the RGB values were used to represent color quantitatively. The RGB values could be converted into visual color, which made it possible to create a color scale that could be used as a reference for grading the quality of rhubarb.

Different concentrations of HAQs display different shades of yellow or red under a neutral or alkaline environment, respectively, which indicates a relationship between the color of the herb and its HAQ content. However, we found that the absolute values of Pearson’s correlation coefficients between the contents of the five HAQs and the RGB values ranged from 0.004 to 0.460 (Table 1), showing non-significant linear correlation between the two variables. It could not be found a direct correlation between the color and quality of rhubarb because the RGB values are distributed in a non-linear space. So, we used artificial neural networks (ANNs), generally developed for non-linear mapping, generalization, self-organization and self-learning [20], to find relationships between the quality and the color of the samples. The most popular method used in ANN-based pattern recognition is the back-propagation (BP) trained, first-order neural network [21], which was used in this study. In Figure 4, it could be found that the outside surface color generated the lowest accuracy compared with the fracture surface color and the powder. After additional color development of the samples with a 0.5% NaOH aqueous solution, the rhubarb displayed a red to dark red color based on the Bornträger reaction of the base with the HAQs. Consequently, the coloration of the samples enhanced the accuracy of the network and increased the number of correct predictions for all sample types. The results suggested that measuring surface color of rhubarb is useless to some extent in assessment of its quality, because the color on the outside surface of the rhubarb can be more easily affected over the course of production and storage than the other sources of color data. The ununiformity was another defect of surface color in assessment of its quality. When the rhubarb is ground into powder form, the uniformity of color can be improved. In addition, developing the color of the HAQs has an advantage in reducing interference from other colored substances. Hence, the color-enhanced powder of rhubarb is preferred in color-based assessment of its quality.

The ANN results indicate that there is a positive but non-linear correlation between the quality of rhubarb and its color. However, the neural network is still a machine-based approach. To facilitate the quality assessment of rhubarb, we tried to establish a colorimetric grading scale to visually discriminate rhubarbs of varying quality. The boundaries of each quality grade of rhubarb were calculated according to their distribution in the chromaticity diagram (Figure 5b). The differences between the colors at the division point (*D*) and the colors of the calculated expected values for each grade can be distinguished with the naked eye, which enables the development of a color scale that can be printed on a card and used to grade rhubarb in the field [22]. For widespread use, the colorimetric grading scale could be printed on paper, similar to professional color cards such as Munsell color cards, to minimize the deviation between the theoretical and individually perceived colors.

In summary, the results of this study revealed that color or color parameters (RGB values) provided important information for the classification of quality of TCMs, such as rhubarb. However, the results varied depending on where the color was sampled: at the surface or fracture surface of the rhubarb, and with or without color development. The BP-ANN network achieved the highest percentage of correct predictions when analyzing powdered samples that were chemically processed to express color, which suggests that powders may be the best type of sample for the accurate grading of rhubarb. On the basis of the distribution of several rhubarb samples in the chromaticity diagram, we have designed a colorimetric grading scale to discriminate between rhubarbs of varying quality. By comparing the color of the sample with the colorimetric grading scale on the card, similar to determining pH using litmus paper, customers without experience in sensory evaluation could quickly assess the quality of rhubarb. This work illustrates the technical feasibility of color-based grading of rhubarb quality and provides useful references for the quantification and standardization of sensory evaluation-based quality control for TCMs, foods and other products.

## Supporting Information

Table S1 **Contents (mg g^−1^) of the five HAQs in the rhubarb samples.**
(DOC)

Text S1 **Ultra Peformance liquid chromotography (UPLC) analysis.**
(DOC)
