# Supplementary material for: Colorimetric Grading Scale Can Promote the Standardization of Experiential and Sensory Evaluation in Quality Control of Traditional Chinese Medicines
Source: PLoS One. 2012 Nov 7;7(11):e48887. doi: 10.1371/journal.pone.0048887 (PMC3492245; doi:10.1371/journal.pone.0048887)
Supplement: Table S1 — Contents (mg g−1) of the five HAQs in the rhubarb samples. (DOC) [file pone.0048887.s001.doc]

Table S1. Contents (mg g^-1^) of the five HAQs in the rhubarb samples [2].

| Sample Nos. | Aloe-emodin | Rhein | Emodin | Chrysophanol | Physcion | Total content |
| --- | --- | --- | --- | --- | --- | --- |
| 1 | 4.41 | 14.58 | 2.91 | 6.99 | 1.71 | 30.60 |
| 2 | 3.94 | 8.46 | 3.35 | 9.93 | 6.04 | 31.72 |
| 3 | 4.11 | 21.08 | 3.58 | 6.10 | 3.62 | 38.49 |
| 4 | 4.32 | 14.11 | 4.34 | 8.91 | 5.63 | 37.31 |
| 5 | 1.80 | 5.33 | 1.71 | 3.81 | 2.06 | 14.71 |
| 6 | 4.57 | 13.14 | 3.40 | 13.47 | 5.54 | 40.12 |
| 7 | 4.54 | 14.72 | 3.34 | 13.32 | 5.45 | 41.37 |
| 8 | 3.07 | 12.15 | 4.81 | 7.18 | 3.94 | 31.15 |
| 9 | 3.19 | 6.93 | 3.48 | 5.16 | 3.44 | 22.20 |
| 10 | 3.98 | 10.79 | 1.98 | 3.77 | 2.33 | 22.85 |
| 11 | 3.50 | 10.66 | 4.68 | 9.61 | 4.59 | 33.04 |
| 12 | 4.36 | 29.84 | 2.35 | 5.10 | 1.98 | 43.63 |
| 13 | 6.57 | 2.63 | 3.39 | 15.80 | 7.64 | 36.03 |
| 14 | 3.41 | 3.82 | 1.27 | 7.00 | 3.51 | 19.01 |
| 15 | 6.32 | 2.63 | 4.65 | 18.43 | 9.76 | 41.79 |
| 16 | 2.15 | 8.37 | 2.05 | 6.70 | 1.58 | 20.85 |
| 17 | 3.18 | 1.39 | 2.26 | 15.81 | 7.11 | 29.75 |
| 18 | 1.69 | 5.21 | 1.68 | 5.58 | 1.44 | 15.60 |
| 19 | 5.55 | 33.48 | 2.97 | 8.55 | 2.52 | 53.07 |
| 20 | 3.01 | 30.65 | 1.66 | 4.78 | 1.12 | 41.22 |
| 21 | 2.94 | 32.37 | 1.99 | 4.51 | 1.27 | 43.08 |
| 22 | 5.63 | 11.63 | 5.01 | 11.15 | 6.12 | 39.54 |
| 23 | 3.11 | 3.04 | 2.01 | 12.77 | 5.75 | 26.68 |
| 24 | 3.48 | 9.65 | 2.48 | 8.68 | 3.36 | 27.65 |
| 25 | 5.71 | 9.30 | 2.27 | 11.52 | 4.91 | 33.71 |
| 26 | 4.92 | 20.12 | 2.54 | 5.19 | 4.13 | 36.90 |
| 27 | 2.55 | 13.45 | 2.55 | 3.36 | 2.02 | 23.93 |
| 28 | 4.40 | 20.45 | 5.38 | 7.39 | 1.13 | 38.75 |
| 29 | 3.53 | 15.02 | 2.09 | 4.19 | 1.55 | 26.38 |
| 30 | 2.39 | 1.71 | 2.10 | 11.45 | 6.48 | 24.13 |
| 31 | 4.00 | 31.62 | 2.24 | 5.32 | 1.96 | 45.14 |
| 32 | 4.03 | 12.61 | 3.03 | 8.48 | 5.04 | 33.19 |
| 33 | 1.66 | 1.01 | 1.86 | 11.90 | 6.72 | 23.15 |
| 34 | 1.92 | 4.56 | 1.66 | 6.26 | 1.81 | 16.21 |
| C.V.(%)^a^ | 34.11 | 74.64 | 38.74 | 46.12 | 57.05 | 29.97 |

^a^ C.V.% =σ/μ×100, where C.V. denotes the coefficient of variance, σ is the standard deviation and μ is the average chemical concentration.
