# Supplementary material for: Colorimetric Grading Scale Can Promote the Standardization of Experiential and Sensory Evaluation in Quality Control of Traditional Chinese Medicines
Source: PLoS One. 2012 Nov 7;7(11):e48887. doi: 10.1371/journal.pone.0048887 (PMC3492245; doi:10.1371/journal.pone.0048887)
Supplement: Text S1 — Ultra Peformance liquid chromotography (UPLC) analysis. (DOC) [file pone.0048887.s002.doc]

**Supporting information**

***Ultra performance liquid chromatography (UPLC) analysis***

*Materials and reagents*

Standard solutions of aloe-emodin, rhein, emodin, chrysophanol and physcion were purchased from the National Institute for the Control of Pharmaceutical and Biological Products of China. HPLC grade methanol was obtained from Fisher Chemicals (Pittsburgh, PA, USA). Water was purified using a Milli-Q water purification system (Millipore, Bedford, MA, USA).

*Standard solution preparation*

Stock solutions of emodin, rhein, aloe-emodin, chrysophanol and physcion (40 μg ml^-1^ of each) were prepared by dissolving accurately weighted portions of the standards in methanol. These solutions were stored in dark glass bottles at 4°C and were stable for at least 1 month. A series of working standard solutions with different concentrations was obtained by diluting the stock solutions with methanol before injection.

*Sample solution preparation*

Powdered rhubarb (0.15 g) was extracted with 25 ml methanol by refluxing for 60 min. The extracted solution was prepared by the weight relief method, where the weight lost in the extraction procedure was compensated. After filtering, 5 ml of the filtrate was transferred to a flask and evaporated to dryness. Subsequently, 10 ml of 2 M HCl and 10 ml of chloroform were added to dissolve the residue. The solution was then kept in a water bath for 1 h. The hydrolyzed solution was extracted in 10 ml of chloroform three times, and the three extracts were combined and evaporated to dryness. The residue was dissolved in methanol and transferred into a 10-ml volumetric flask. The solutions were filtered through a filter with 0.22 μm pores before injection.

*Chromatographic conditions*

UPLC was performed using a Waters Acquity system equipped with a binary solvent delivery pump, an auto sampler and a photo diode array detector. Chromatographic separation was performed using a Waters Acquity BEH 50 mm×2.1 mm, 1.7 μm, C_18_ column. The mobile phase was a mixture of 0.1% aqueous phosphoric acid and methanol in the ratio of 36: 64 (v/v) and was introduced at a flow rate of 0.65 ml min^-1^. The detector wavelength was set at 254 nm. The injection volume was 1 μl, and the temperature of the column was maintained at 35°C. The UPLC method was validated in the previous paper [1].

*Contents of the five HAQs in the samples*

The contents of the five HAQs in the thirty-four rhubarb samples [2] are shown in Table S1.

*References:*

[1] Wang JB, Li HF, Jin Cheng, Qu Y, Xiao XH. Development and validation of a UPLC method for quality control of rhubarb-based medicine: Fast simultaneous determination of five anthraquinone derivatives. Journal of Pharmaceutical and Biomedical Analysis, 2008, 47(4-5): 765-770.

[2] Wang JB, Zhang XR, Xiao XH, Chu XH, Zhou CP, et al. Rationality of commercial specification of rhubarb based on chemical analysis. China Journal of Chinese Materia Medica, 2010, 35(4): 470-476.
